# Therapeutic Strategies for Neuropathic Pain: Potential Application of Pharmacosynthetics and Optogenetics

**DOI:** 10.1155/2016/5808215

**Published:** 2016-01-13

**Authors:** Gum Hwa Lee, Sang Seong Kim

**Affiliations:** ^1^College of Pharmacy, Chosun University, Gwangju 501-759, Republic of Korea; ^2^Department of Pharmacy, Hanyang University, ERICA Campus, 55 Hanyangdaehak-ro, Sannok-gu, Ansan, Gyeonggi-do 426-791, Republic of Korea

## Abstract

Chronic pain originating from neuronal damage remains an incurable symptom debilitating patients. Proposed molecular modalities in neuropathic pain include ion channel expressions, immune reactions, and inflammatory substrate diffusions. Recent advances in RNA sequence analysis have discovered specific ion channel expressions in nociceptors such as transient receptor potential (TRP) channels, voltage-gated potassium, and sodium channels. G protein-coupled receptors (GPCRs) also play an important role in triggering surrounding immune cells. The multiple protein expressions complicate therapeutic development for neuropathic pain. Recent progress in optogenetics and pharmacogenetics may herald the development of novel therapeutics for the incurable pain. Designer Receptors Exclusively Activated by Designer Drugs (DREADDs) facilitate the artificial manipulation of intracellular signaling through excitatory or inhibitory G protein subunits activated by biologically inert synthetic ligands. Expression of excitatory channelrhodopsins and inhibitory halorhodopsins on injured neurons or surrounding cells can attenuate neuropathic pain precisely controlled by light stimulation. To achieve the discrete treatment of injured neurons, we can exploit the transcriptome database obtained by RNA sequence analysis in specific neuropathies. This can recommend the suitable promoter information to target the injury sites circumventing intact neurons. Therefore, novel strategies benefiting from pharmacogenetics, optogenetics, and RNA sequencing might be promising for neuropathic pain treatment in future.

## 1. Introduction

Pain is an unpleasant sensory and emotional experience associated with actual or potential tissue damage [1, 2]. Extreme pressure, abnormal temperature, or pH elicits a pain sensation in the brain, following pain avoidance behavior. Therefore, appropriate and prompt pain sensation is critical to adapt to the outside environment. Neural pathways transmitting pain in the peripheral and central nervous systems are well characterized. Transducers on primary afferent sensory nerve terminal, including transient receptor potential (TRP) ion channels, convert painful chemical or mechanical stimuli into electrical signals, thereby initiating activation of sensory nerves [3, 4]. Action potential initiated in primary afferent nociceptors is transmitted to higher sensory brain cortex via multiple synaptic connections in the central nervous system. Pain signal transmission is regulated by the vast complexity of excitatory and inhibitory neural networks and ligand-receptor pairs [5–7], indicating the importance of proper regulation and adaptation of these sensory modalities to maintain homeostasis of our body response to external painful stimuli.

In disease, prolonged tissue damage or inflammation induces alterations of gene expression and membrane protein modifications in such a way that aberrant activation of certain nociceptor occurs, even in the absence of noxious stimuli [8–10]. Millions of people worldwide suffer from chronic pain with lack of proper analgesic treatment options [8]. Based upon our knowledge of cellular modalities including primary afferent nociceptors, immune cells, and glial cells and molecular entities including ion channels, G protein-coupled receptors, neurotransmitters, inflammatory mediators, kinases, and growth factors involved in pain transmission, several target-based pharmacological approaches and anti-inflammatory drugs have been partially successful in controlling several pain symptoms [5, 6, 8, 11]. However, when pain is originated from dysfunction in relaying pain signals from the sensory nerve or neuron network to the central nervous system, it is difficult to control pain transmission with a conventional target-based approach. This is mainly because multigenic origin and multiple cellular and molecular targets are involved in the development of neuropathic pain. Whole genome RNA sequencing (RNAseq) or microarray explorations of neuropathic pain have revealed dramatic and extensive changes in gene expression profile during the disease process [12–17]. Moreover, it is difficult to diagnose the exact cause underlying ongoing pain symptoms. The highly plastic nature of neurons and their surrounding environments could be an obstacle for conventional single target-based therapeutic strategy in controlling pain, necessitating the novel therapeutic approaches that are more selective and systemic in their effect.

Control of neural activity using light activated ion channels (optogenetics) or using designer receptor G protein-coupled receptors (GPCRs), abbreviated DREADDs, has become possible and has been tested in several animal models. Since neuropathic pain is attributed largely to the abnormal activity of neurons or immune cells, it could be possible to employ these techniques in suppressing abnormal pain signal transmission. In this review, we discuss molecular and cellular changes in neuropathic pain development with the main focus on peripheral sensory nerve. This review highlights the use of optogenetics and DREADDs in controlling neuronal activity and elaborates the advantage and possibility of their application in the treatment of neuropathic pain.

## 2. Molecular Mechanism of Neuropathic Pain

Pain is a subjective sensation in response to obnoxious stimuli that include toxic chemicals, mechanical stress, extreme temperature, and tissue damage [1]. External pain stimuli are first detected at cellular levels at primary afferent sensory neurons in the dorsal root ganglion (DRG). Peripheral pain sensory nerves are structurally distinct from other nerves. They are termed nociceptive C fibers and are characterized by a small diameter cell body and unmyelinated axons [18].

Neuropathic pain is defined as a pain state arising from neuronal lesion, which alters the degree of pain [19, 20]. Neuropathic pain is experienced by millions of people globally. Current treatment options only provide about 50% relief for only 40–60% of patients [20]. Complaints include spontaneous pain, burning pain, or tingling and exaggerated responses to innocuous and noxious mechanical or thermal stimuli that include allodynia, hyperalgesia, and hypersensitivity to heat or cold. Neuropathic pain develops as a consequence of dramatic changes in multiple levels of nociceptive pain wires following formation of a nerve lesion. The cause of neural damage can be diverse and includes traumatic injury, inflammation, and metabolic diseases like diabetes, toxins, viral infections, or malignant cancer. The alterations in response to nerve lesions are extensive and encompass gene profile changes with novel receptor and ion channel presentation at the membranes, alterations of nociceptive signal processing in the peripheral and central nervous system, and finally pathologic synapse formation with neighboring neurons [9, 10, 21–23].

Substances released in the vicinity of spared fibers by Wallerian degeneration (e.g., nerve growth factor) or calcium influx into neurons may underlie substantial transcriptome remodeling in neuropathic pain development [10]. Hypersensitization of primary afferent nociceptors contributes to pathological pain sensation (hyperalgesia or allodynia). Hyperactivity input from peripheral neuropathic nociceptors in turn leads to central sensitization that interprets mild stimuli from periphery, such as touch or proprioception, as pain sensation [21].

### 2.1. Nociceptor Alterations during Neuropathic Pain Development

**Figure 1:**
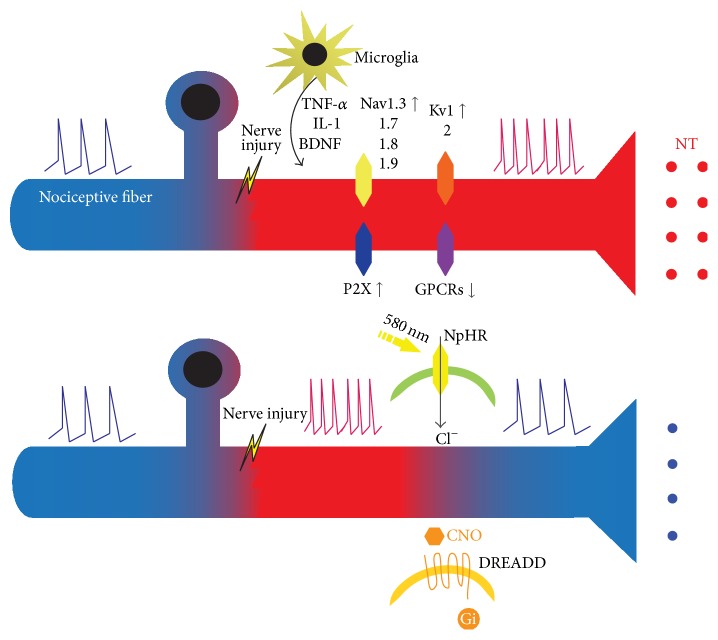
Illustration of molecular changes contributing to neuropathic pain in nociceptive fiber and potential strategy to suppress the hyperexcitability. Following nerve lesion or inflammation in nociceptive fiber, nociceptive sensory afferent undergoes chronic alteration in expression profiles of membrane proteins. Inflammatory mediators secreted from activated immune cells can initiate transcriptional remodeling on DRG nociceptors by acting on G protein-coupled receptors (GPCRs). Alterations of nociceptor membranes in neuropathic pain development involve upregulation of several subtypes of voltage-gated sodium channels and downregulation of membrane stabilizing voltage-gated potassium channels. These alterations on the excitable membrane lead to the exaggerated action potential propagation in response to subnoxious stimuli, ultimately transmitting neuropathic pain perception in higher brain structure. The abnormal action potential firing in neuropathic pain-inflicted DRG could be suppressed via expression of halorhodopsin or inhibitory G protein-coupled DREADDs, both of which can stabilize membrane potentials. Application of light or CNO for these types of membrane proteins could provide opportunity to control neuropathic pain transmission immediately.

Nerve lesion-triggered molecular changes in neuropathic pain remodel nociceptors. The remodeling can cause abnormal sensitivity and can generate pathological spontaneous activities (Figure 1). Expression of diverse membrane proteins is altered, which may contribute to hypersensitization of nociceptive neurons. Several independent studies and a recent global RNAseq analysis revealed that pain sensing TRP ion channel expression and voltage-gated sodium channel subtypes Nav1.7, Nav1.8, Nav1.9, and Nav1.3 are increased in nociceptors in neuropathic pain models [6, 14, 16, 23]. In the normal condition, functional potassium currents provide inhibitory control over neuronal activity and are critical for preventing ectopic activity of neurons. Along with ion channel expression contributing to membrane depolarization, membrane stabilizing potassium channel Kv1 and Kv2 family subunits are impaired in pathological conditions, such as experimental diabetes [5, 6]. Moreover, Kv7 (KCNQ) contributes to generation and maintenance of neuropathic pain [24, 25]. All of these changes lower the threshold for nociceptor activation. In this condition, even mild stimuli can be sufficient to elicit action potential in nociceptive neurons. In other words, activation of even a few nociceptors can be sufficient to transmit high frequency pain signal to the sensory cortex.

### 2.2. Immune Cell Activation during Neuropathic Pain

Together with nociceptors, several types of immune cells are involved in the development and maintenance of neuropathic pain. Immune cells in peripheral injury sites include mast cells, neutrophils, monocytes, and macrophages. The role of immune cells in sensitizing nociceptors and their involvement in maintaining neuropathic pain are thoroughly reviewed elsewhere [9, 26]. The inflammatory mediators released from immune cells can directly act on nociceptors initiating pain signaling cascade [9, 10]. Notably, diffusible inflammatory mediators and substances from damaged neurons affect adjacent uninjured neurons, spreading pathological changes to a broader area. Experiments that restricted the recruitment of these immune cells to neuronal injury sites have shown protective effects against development of neuropathic pain in animals [11, 27], demonstrating the important role of immune cell-nociceptor interplay in the disease progression. Imbalance between proinflammatory cytokines and anti-inflammatory cytokines has been found in neuropathic pain patients [28, 29]. Interestingly, animal models of neuropathic pain have demonstrated upregulation of proinflammatory cytokines, such as TNF-*α*, IL-1*β*, and TNF receptors [30–32]. Infusion of these cytokines is sufficient to induce neuropathic pain development [33, 34], indicating a role of immune cells in the early stage of neuropathic pain development.

### 2.3. GPCRs in Neuropathic Pain Development

GPCRs also play important roles in the process of nociceptor sensitization. In contrast to short-acting ion channel function, GPCR can mediate long-lasting changes in gene expression or membrane excitability [35, 36]. Similar to ion channels, GPCRs also undergo substantial changes in expression during neuropathic pain development. Nerve lesions lead to induction of *α*1 or *α*2 adrenoceptors on cutaneous afferent fibers, driving the development of neuropathic pain associated with and responding to sympathetic stimulation [37, 38]. In some neuropathic patients, sympathetic efferent may cause excitation of nociceptors by noradrenaline or circulating catecholamines with additional expression of *α*1 receptors, initiating pain associated with sympathetic stimulation. Immune cell activation involves many GPCRs for classic chemoattractants and chemokines [10, 35]. Notably, the activation of GPCR often leads to fundamental transcription changes or inflammatory mediator release through divergence of signaling cascades. During inflammation, a variety of GPCR agonists act on GPCRs on nociceptors leading to hypersensitivity (Figure 1). Bradykinin and some prostaglandins are upregulated during the initial stage of neuropathic pain through interaction with B1 and B2 bradykinin receptors or nine prostanoid receptors that are coupled with diverse G proteins [11]. During nociceptor sensitization, GPCRs for prostaglandin or substance P mediate intracellular signaling cascades that result in posttranslational modification of sodium or N-methyl-D-aspartic acid (NMDA) receptors leading to hypersensitization of nociception in DRG and the CNS [20, 39].

## 3. Pitfalls of Conventional Neuropathic Pain Treatment and Novel Techniques as Therapeutic Candidates

As demonstrated by microarray and RNAseq whole transcriptome analysis, extensive changes of gene expression underlie structural and functional remodeling of nociceptors in pathological neuropathic pain [14, 17, 23]. In this regard, conventional strategies to inhibit individual ion channels or inflammatory processes have not been useful, due to the complex etiology of the pathological changes. Moreover, commonly used pain medications including opioid analgesics or NSAIDs oftentimes develop side effects and tolerance. Supporting the complicated molecular mechanism of neuropathic pain, Nav1.3 or Nav1.8 knockdown or knockout mouse models still develop neuropathic pain, although their contribution to hypersensitization of nociceptive neurons has been amply demonstrated* in vitro* [40, 41]. Considering the extensive isoforms of sodium channels or other receptor subtypes that are responsible for hypersensitization, selective inhibitors should be administered to minimize adverse side effects [6]. However, the availability of those selective therapeutic ligands is limited. Centrally acting drugs like antidepressants, anticonvulsants, and opioids provide partial pain relief, but the treatment of neuropathic pain is still unsatisfactory [8, 19]. Moreover, these centrally acting drugs are lipophilic and nonspecific with a narrow therapeutic window, further preventing their prolonged use for chronic pain treatment. Treatment of chronic pain requires a novel approach regulating excitability of nociceptors as a final outcome, since it is clear that nociceptors and immune cells behave abnormally in neuropathic pain. Genetic approaches that target these activated neurons in selective manner could be a potential therapeutic strategy.

### 3.1. Designer Receptors Exclusively Activated by Designer Drug (DREADD)

Pharmacosynthetics and chemogenetic tools modulate transgenic receptors using artificial pharmacologic agents [42]. Currently, the most plausible pharmacosynthetic tool is the Designer Receptors Exclusively Activated by Designer Drug (DREADD) [43]. DREADDs are activated only by synthetic ligands like clozapine N-oxide (CNO), which are otherwise an inert pharmacological agent. DREADDs are GPCRs capable of being pharmacologically modulated by synthetic chemicals, but not by endogenous GPCR ligands. A DREADD receptor can be engineered to be coupled with G*α*s (rM3Ds), Gi (hM4Di), or Gq (hM3Dq) [42] to activate different secondary signaling pathways and different modulation of neuronal or cellular functions [42]. Transgenic expression of DREADDs in specific cell types enables selective modulation of these DREADD expressing cells by ligands. Except for the fact that they can be controlled with chemical ligands, DREADDs behave like other endogenous GPCRs, interacting efficiently with downstream intracellular signaling components. For example, one study employed rM3Ds DREADDs to study the role of G*α*s/cAMP/protein kinase K signaling cascades in the striatal medium spiny neurons for the hyperlocomotor response following psychostimulant treatment, such as methamphetamine or cocaine treatment [44]. To establish DREADDs* in vivo*, transgenic mice expressing rM3Ds in medium spiny neuron (MSN) were generated using the promoter adenosine A2A. With rM3Ds expression in MSN, CNO treatment inhibited hyperlocomotor response even in the presence of amphetamine, thus demonstrating the important contribution of GPCR signaling in MSN for this type of behavior [44]. In addition, a novel Gi-coupled DREADD has been recently developed by reengineering kappa-opioid receptor as a template [45]. This inhibitory DREADD is activated pharmacologically by the inert ligand, salvinorin B (SALB). The authors reported that M3-DREADD multiplexed chemogenetic regulation was controlled by CNO.

**Figure 2:**
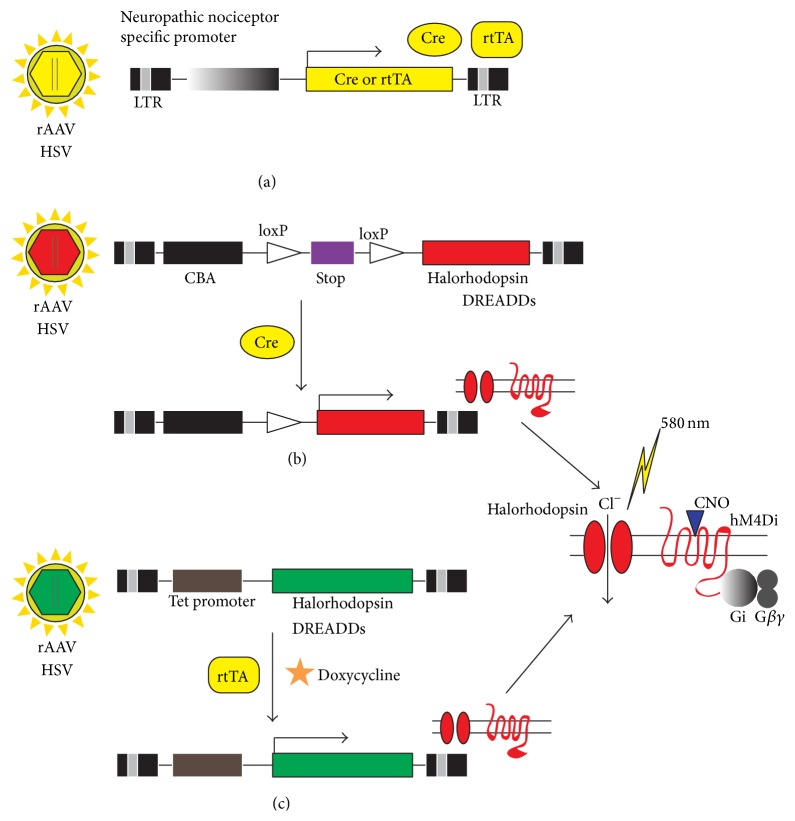
Schematic strategy to achieve cell type specific application of optogenetics and DREADDs for the treatment of neuropathic pain. (a) To realize optogenetic- or DREADD-mediated stabilization of aberrant nociceptor activity in neuropathic pain, two viral vector systems can be employed. Relatively neuron selective serotypes of recombinant adeno-associated virus (rAAV) or herpes simplex virus (HSV) can be used to deliver viral constructs to dorsal root ganglion. To achieve conditional expression of channel rhodopsin or DREADDs, Cre recombinase or reverse tetracycline activated transcriptional activator (rtTA) is expressed under the control of promoters which are selectively activated in neuropathic pain affected nociceptors. (b) In case of the Cre-loxP system, viral expression of halorhodopsin (yellow light-gated chloride ion channel, eNpHR3.0) is prevented by loxP flanked transcription stop cassette (STOP) downstream of the strong promoter chicken beta actin (CBA). Transgene expression is only turned on in cells where Cre recombinase is expressed and STOP cassette is removed. (c) In Tet-On system, transgene expression is under the control of tetracycline responsive promoter (Tet promoter). Transgenes begin to be expressed in rtTA expressing nociceptors only when doxycycline is administered. Transgene expression can be controlled reversibly by withdrawing doxycycline. Following region specific expression of halorhodopsin or DREADDs in nociceptor, membrane stabilization can be induced by halorhodopsin activation by 580 nm light stimulation or CNO binding to inhibitory hM4Di-DREADDs.

In addition, GPCRs have a diverse expression pattern in tissues and they are the most popular drug targets, representing 36% of currently approved drug targets [42]. For this reason, DREADDs draw attention for the potential in the treatment of pathological conditions that involve abnormal GPCR downstream signaling. GPCR is a naturally occurring metabotropic receptor that mediates diverse neuronal functions including gene expression. GPCR often collaborates with ion channels leading to activation or inhibition of ion channel activity. Therefore, appropriate DREADD expression in neurons can counteract aberrant neuronal excitability through long-lasting secondary changes to an excitable membrane. For instance, the expression of hM3Dq DREADD on neurons and activation by CNO can lead to effective membrane depolarization, whereas hM4Di expression inhibits neuronal action potential firing (Figures 1 and 2).

### 3.2. Optogenetics for Neuropathic Pain Treatment

Optogenetics utilizes channel rhodopsin that can be opened by certain light frequency stimulus, thus controlling neuronal firing [46]. Chimeric ion channels expressed in specific cell types by the conditional transgenic approach enable selective stimulation of certain neuronal population with fiber optics inserted into the desired site to deliver light to neurons. Channelrhodopsin (ChR) is engineered to be selective for cation entry whereas halorhodopsin (NpHR) is selective for anion flux so that the activity of neuron can be effectively controlled by either stimulation or inhibition as intended. Combined with the conditional transgenic approach that expresses it in specific neuronal circuit, optogenetics has been extensively used to delineate neuronal connection and pathways even in the control of emotion or behavior [46, 47].

In terms of pain sensation, optogenetics has been employed to elicit pain with a stimulatory opsin expression in Nav1.8 positive neurons in combination with transdermal optogenetic activation [48]. In other cases, it also mitigate neuropathic pain with an inhibitory opsin expression in sensory nociceptive neuron population via adeno-associated virus (AAV) serotype 6 infection and expression of opsin by neuron specific human synapsin-1 promoter [49]. Pain modulation by optogenetics* in vivo* has been successfully attempted in controlling neuropathic pain [49]. AAV6 virus expressing ChR has been delivered to sensory neurons by the intrasciatic injection. This delivery route allows very efficient access in small diameter nociceptive nerves salvaging large-diameter myelinated neurons that transduce touch and proprioception [50]. Optogenetic inhibition of action potential generation in nociceptors has been attempted using yellow light sensitive third-generation chloride pump halorhodopsin (eNpHR3.0). When delivered via AAV6 intrasciatic injection, eNpHR3.0 activation in DRG neurons with constant yellow light illumination strongly inhibits action potential initiation by membrane hyperpolarization.* In vivo* stimulation of this channel was sufficient to prevent painful behavior in neuropathic pain model, suggesting its therapeutic potential [49].

### 3.3. Comparison between DREADDs and Optogenetics Application

The rationale of applying optogenetics or DREADDs for control of abnormal pain perception in neuropathic pain is straightforward. In neuropathic pain, pain is heightened by the complicated modification of structure and gene expression in nociceptors. In this condition, neurons are easily activated to generate action potentials and transmit signals through an already built-in pain transmission network wired to the sensory cortex. Optogenetics modulates neuronal activity by regulating channel opening with controllable light application. As demonstrated by* in vivo* research discussed in the previous section [49], dampening of excitation in the pathologically sensitized nociceptors could be possible by expressing halorhodopsin for Cl^−^ influx, thereby stabilizing membrane potential to prevent further high frequency action potential generation (Figure 1). Discrete expression of halorhodopsin in nociceptors is crucial to achieving selective inhibition in sensitized nociceptors. On the other hand, if interneurons are synapsed with pathological nociceptors, excitable opsins can be also employed together in the interneurons to enhance inhibitory capacity by flowing abundant inhibitory neurotransmitters, such as GABA and glycine, at certain levels. Similarly, Gi-coupled DREADDs could be expressed in injured nociceptors to induce inhibitory changes on the membrane excitability. Gi-coupled inhibition of adenylyl cyclase and resulting decrease of cAMP and PKA signaling pathway can reverse pathological changes that have already occurred in neuropathic pain (Figure 1).

The difference between the optogenetics and DREADD approaches could be the duration of the effects and the extent of the downstream influence. Optogenetic-mediated nociceptor inhibition can be directly achieved by fast-acting membrane potential stabilization, which occurs within a few milliseconds after light signal is applied. On the other hand, DREADDs involve second messenger activation and, sometimes, alteration of gene expression changes [42, 45, 51]. This can cause longer latency time to achieve actual therapeutic effect, but this effect could be longer-lasting. Many therapeutically effective chemicals are modulators rather than direct ligands, which inhibit or open ion channels [52]. In this respect, the use of DREADDs could be a relatively safer approach in actual human trials compared to optogenetic modulation of nociceptors, which could have potential adverse effects.

Extensive expression profiles with various physiologic involvements of GPCRs also favor application of DREADDs in pain modulation. GPCR is involved in nociceptive neurons and in the activation and functioning of immune cells [9, 10]. Thus, DREADD expression in regions where neuropathic pain develops is able to control aberrant activation of immune cells, which ultimately induces and maintains the hypersensitivity of nociceptors. Since immune cells propagate pathological chemokines and inflammatory mediators, pharmacological control of stimulated immune cells by DREADDs, together with control of nociceptor activity, will facilitate more successful control of neuropathic pain transmission.

### 3.4. Temporal and Spatial Selectivity

A characteristic of neuropathic pain development is that the inflicted neurons and pain pathways are highly restricted. Drug treatment in itself has intrinsic weakness in the lack of selectivity, which can cause unwanted adverse effect, especially at higher doses. In this respect, DREADDs can be only activated by specific ligands that otherwise do not interact with other endogenous receptors at the therapeutic dosage range [42]. As long as DREADDs are expressed in the desired region, the intended effect can be exerted in specific site. In similar way, the optogenetic approach also provides spatial selectivity. Conditional expression of ChR can be achieved with tissue specific promoters or by delivery of channel genes via stereotaxic viral injection to target regions (Figure 2). Light-gated halorhodopsin or inhibitory DREADDs can be also combined with conditional approaches to enhance cell type specific expression (Figure 2). By combining double-floxed inverted opsins [53] with AAV expressing Cre recombinase under the control of tissue specific promoter, it could be plausible to drive inhibitory opsin expression in desired neuronal or pathologically inflicted neurons to control pain signal transmission to higher structure of pain perception [53]. As an exemplary application of Cre-mediated DREADD expression in specific neuronal population [53], hM3D1 or hM4Di DREADD has been expressed in agouti related protein (AgRP) expressing neurons by using a Cre-loxP system [51]. When loxP-blocked hM3Dq AAV was injected into AgRP-IRES-cre mice, hM3Dq expression was only confined to cell types expressing cre under control of the AgRP promoter. Interestingly, depending on G protein signaling modulated with CNO (0.3 mg/kg) administration, the eating behavior of the mice was reversibly controlled, demonstrating the role of G protein-coupled signaling in this type of neural circuit for food intake behavior [51].

Temporal regulation is also possible because DREADDs or ChRs can be only activated when the corresponding stimuli are given either by pharmacological application of ligands or by light stimuli in the vicinity. Withdrawal of drugs or light can terminate activity of these neurons. While the ChR response upon withdrawal of light is immediate, DREADD activity will be gradually reduced following pharmacokinetic elimination of ligand resulting in longer-lasting changes. In this respect, the optogenetic approach manifests better temporal resolution in modulating nociceptor activity.

The tet-on system is applied for controlled expression spatially and temporally by relaying actions of rtTA acting on tetO promoter [54]. When rtTA expression is driven by tissue specific promoter, tetO-driven DREADD transgene expression can be restricted to specific tissue. Reversible expression of DREADD receptor is achieved by doxycycline dependent activation of rtTA action on tetO promoter. By combining with DREADD-CNO coupling, the tet-on system will provide additional safeguard to control DREADD expression as well as DREADD-mediated G protein signaling in targeted cell population (Figure 2).

## 4. Optimization for Advanced Therapeutic Methods to Attain Selectivity

Viral delivery is a very efficient tool to deliver any gene of interest* in vivo* [55]. Although we are at mostly preclinical validation stages of viral gene therapy for neuropathy pain control, there has been great success in suppressing pain sensation by viral transfer of single gene in neuropathic animal models [56]. Due to the efficient delivery of therapeutic genes and promising preclinical results in several disease animal models, clinical trials for this method have been made for over 20 years.

To increase safety and selectivity of clinical application of the viral vector-mediated gene therapy, the following issues need to be addressed [57]. Firstly, the viral vector should be devoid of replication ability, genomic integration, and immunogenicity to be safely applied to human subjects. Secondly, the neuropathic disease states are usually chronic, persisting for long period of time. This makes it necessary for the viral vector to maintain constitutive expression levels in therapeutic dosage. Thirdly, gene delivery should be targeted to specific cells to prevent undesirable toxicity from transgene expression in other areas with optimal titer volume. Some candidate viral vectors fall into the categories such as adeno-associated virus (AAV), herpes simplex virus (HSV), and lentivirus [56]. These viral vectors offer attractive features like less immunogenicity and genomic insertion possibility than other viral vectors. Indeed there have been ongoing clinical trials with HSV and AAV to treat inherited disease with defective gene expression [57–61]. Lentiviral vector has advantage to harbor larger transgene in the virus particle and it shows tropism for neuron transduction, but its genomic integration makes it relatively unsafe [62]. Its application is mostly confined to* ex vivo* gene transfer. But still there has been an attempt to apply this virus for neurodegenerative diseases such as Parkinson's disease. On the other hand, HSV and AAV seldom integrate into host genome and they have tropism for neuron transduction depending on serotypes used for gene delivery [56]. These features and others provide advantages in translational research and clinical application of the viral vectors for neuropathic pain modulation.

### 4.1. Clinical Application of AAV-Mediated Gene Therapy

AAV virus has advantage compared to other viral vectors. For example, the long term expression is possible via epitome stabilization. Genomic integration seldom occurs, preventing insertional mutation and tumorigenesis [56]. The wild type AAV is not causing any known disease in human, confirming safety in practice. Small size of AAV with diameter of 20–25 nm enables highly robust diffusion even in application to solid tissues. It can still encompass up to 5 kb size of DNA into virus particle which is sufficient to clone gene of interest for delivery. Several reports have shown efficient transduction of several cell types including neurons* in vivo* animal studies and clinical trials [57]. Due to these attractive features of AAV virus, AAV-mediated gene therapy has been extensively studied, and long term expression of genes for therapeutic purpose has been achieved in a clinical setting especially for patients with inherited disorders such as retinal disorders and hemophilia B [57, 59]. Although successful application of viral gene therapy varied depending on target tissues and replacement genes of interest, there have been reports demonstrating safety and therapeutic efficacy of AAV-mediated gene transfer to patients with inherited disease defective in certain genes.

As mentioned above, AAV can transduce dividing and postmitotic tissues with specificity depending on its serotypes. AAV serotype 2 has been widely used since it shows relative selectivity for neuron transduction in comparison to glial transduction, while AAV serotype 1 tends to infect both neurons and glial cells in nonspecific manner [56]. Thus, depending on target cells of interest, appropriate AAV viral serotypes can be chosen for best result. Actually, in animal model studies, AAV has been successfully administered via intrathecal or direct injection to DRG to achieve transduction of afferent sensory neurons and glial cells. Serotype 1 AAV, on the other hand, has tendency to transduce not only neurons but glial cell population; it would be possible to transfer inhibitory DREADDs to inflammatory glial cells that are highly activated in neuropathic pain environments.

However, there are certainly disadvantages in AAV application, too. Even though its sizable capacity is up to 5 kb of DNA into the virus particle, it would be difficult to package cell type specific promoter components that are usually bigger than 5 kb [56]. Therefore, it would be hard to purify AAV virus expressing channel rhodopsin or DREADDs via responsive promoter (e.g., tet promoter, or loxP flanked STOP cassette containing CBA promoter). It may be necessary to use combinatorial viral application using other viral vectors that can hold larger capacity to package bigger size of foreign DNA like HSV vector. Furthermore, AAV clinical application could induce adverse immune reactions through AAV viral capsid responsive T cell activation or transgene expression itself. So caution should be taken to monitor safety and effectiveness of AAV viral application.

### 4.2. Clinical Application of HSV Mediated Gene Therapy

Replication-defective herpes simplex virus (HSV), thus nonpathogenic, has been used for gene therapy in animal models and human clinical trials. The clinical trial of nonreplicating HSV expressing preproenkephalin treatment for intractable pain in cancer patients has shown to be safe with dose responsive therapeutic efficacy to relieve pain, providing proof of evidence for gene therapy with HSV virus [61, 63]. Different from AAV approach, HSV has a tendency to transduce neurons in a retrograde manner, which makes this vector suitable for targeting afferent sensory nerves [63]. HSV vectors expressing antinociceptive substances, such as endomorphin-1, proenkephalin A, and IL-4, have been subcutaneously inoculated to selectively transduce DRG in neuropathic animal models [56].

Most striking feature of HSV vector is that it has strong tropism for nerve cells and high capacity to contain sizable DNA fragment. Furthermore, once introduced into neurons via retrograde transport, the viral genes are stable and exert sustained expression of transgenes. There features are quite advantageous in clinical application for human neuropathy because conditional DREADDs and halorhodopsin gene expression in disease specific manner requires selection and usage of tissue specific gene promoter that is usually big in size ranging around 10 kb. Thus HSV viral vector is suitable to transfer neuropathic nociceptor specific promoter driving expression of Cre or rtTA into abnormally activated nociceptors with sustainable expression of transgenes (Figure 2).

Despite some promising clinical application of viral vector-mediated gene therapy, caution should be taken because unexpected toxicity due to irreversible expression of therapeutic genes could be harmful to patients. In this respect, our proposed conditional viral model system to deliver either Cre responsive or tetracycline responsive halorhodopsin or inhibitory DREADDs provides additional safeguard. Different from previously attempted constitutive expression of antinociceptive proteins, expression of halorhodopsin or inhibitory DREADDs is switchable even after successful integration into neuropathic pain sites. As discussed above, channelrhodopsin and DREADDs retain temporal resolution in their activation and function via lighting or CNO ligand treatment, respectively. Any unwanted adverse effects after clinical application can be immediately abolished by simply withdrawing the aforementioned gating stimuli. In addition, tet-off controlled expression of channelrhodopsin or DREADDs (Figure 2(c)) allows stopping expression of this transgene itself. Thus, if transgene expression initiates harmful immune response in clinical setting, the expression of therapeutic genes can be turned off by withdrawing doxycycline treatment.

Overall, the safety and efficacy of gene therapy mediated treatment for clinical neuropathy could be improved substantially by combining optogenetics/DREADDs, conditional genetic tools, and appropriate viral vectors.

### 4.3. Gene Expression Profiling during Neuropathy

Tissue type selective expression and temporal control of ChRs or DREADDs may not be sufficient to pinpoint the damaged nociceptive neurons. To gain specific control over neuropathic pain, it is essential to understand which genetic program is differentially activated in these pathological neurons compared to normally functioning neural circuits. Differential promoter activities between normal and neuropathic states can be exploited to construct transgenic viral vectors for DREADDS or ChRs to be expressed in more defined pathological cell types (Figure 2). Rather than affecting normal pain pathways, this approach will ensure disease specific control for drug treatment. As discussed above, normal sensation of pain is crucial to adapting to outside danger through proper response [18, 64]. The selective expression of therapeutic modules in abnormally stimulated nociceptive neurons with altered gene expressions will attenuate abnormal pain sensation while preserving normal sensation of pain.

To accomplish this, a complete understanding of global gene expression profiling is needed. Previously, microarray with probes printed on slides was a representative method to obtain transcriptome profiling. Microarray experiments are relatively easy to perform and are adequate for high-throughput data acquisition. There are many microarray databases pertaining to neuropathic pain that have provided insight into differentially regulated genes in neuropathic pain state [23, 65]. These have shown that several neuropeptide messenger levels (NPY, galanin, and VIP) are highly elevated in DRG of neuropathic pain models. Inflammatory mediators such as complement proteins, allograft inflammatory factor-1, alpha-2-macroglobulin, interferon-induced guanylate-binding protein 2, and IL-18 are also upregulated. Notably, upregulated receptors include GABA receptor, nicotinic acetylcholine receptor *α*7 subunit, P2Y1 purinoceptor, Na channel *β*2 subunit, and Ca channel *α*2*δ*-1 subunit [23, 65], providing ample promising promoter candidates for selective induction of DREADDs or ChRs.

Microarray-based transcriptome analysis often has shortcomings due to the limited pool of custom-designed probes for gene detection. Furthermore, depending on probe and messenger pairing, absolute comparison of each gene expression level is almost impossible, only detecting relative changes of certain gene expression. On the other hand, RNAseq technology enables unprecedented global characterization of transcriptome changes* in vitro* and* in vivo* with greater sensitivity. Due to its wide detection range, RNAseq provides quantitative values (e.g., reads/fragments per kilobase of transcript per million mapped reads) that can be used to estimate gene abundance [66]. It is also possible to do RNAseq transcriptome profiling if it is combined with BAC-TRAP technology, even in brains with highly heterogeneous distribution of neuronal and glial cell types [67].

RNAseq in DRG neurons has been described in normal [15] and pathological neuropathic pain condition [14]. This global profiling can be used as a basis to identify neuropathic pain specific promoters that are highly activated in nociceptors. Several genes with more than 10-fold enrichment in DRG have been identified in this global RNAseq analysis. These data can be utilized to mine sensory neuron specific promoters for disease modifying transgene. Even in ultraviolet-induced inflammatory pain, RNAseq analysis in damaged DRG has successfully identified upregulated expression of REG3B, CCL2, and VGF [14]. Application and comparison of RNA whole transcriptome sequencing in defined neuropathic pain animal models or in patient samples will provide detailed and systemic molecular insights underlying the disease process.

Combined with the genetic engineering and functional expression of DREADDs and ChRs, better strategy for neuropathic pain control could be designed as compared to pan neuronal expression of these modules, which could shut down the overall pain sensation, possibly leading to adverse effects.

## 5. Conclusions

Neuropathic pain involves complicated and diverse molecular changes in nociceptive neurons. Since conventional therapies targeting single target genes including ion channels, receptors, or inflammatory mediators have been largely unsatisfactory, novel therapeutic strategies are needed. Optogenetics and DREADDS provide hope in controlling intractable pain in patients suffering from debilitating neuropathic pain. These tools can provide an effective means to control the disease process by artificial drugs through the modulation of distinct neuronal populations. Based upon the underlying cause of neuropathic pain, it would be possible to improve the potential of pain management by combination therapies of these techniques and drugs targeting specific molecular targets. With continuing development and refinement of this technique in combination with viral vector-mediated safe and efficient gene therapy, it is our hope to develop satisfactory treatment for otherwise intractable neuropathic pain.
